# Supplementary material for: Modeling glioblastoma heterogeneity as a dynamic network of cell states
Source: Mol Syst Biol. 2021 Sep 16;17(9):e10105. doi: 10.15252/msb.202010105 (PMC8444284; doi:10.15252/msb.202010105)
Supplement: Supplementary file 6 — Source Data for Figure 5 [file MSB-17-e10105-s004.zip › Figure5A_sourcedata/GSEA_3071/nosigdb_stateII.GseaPreranked.1622795151171/RICHARDS_NOWAKOWSKI_SCIENCE_2017_NIN3_UPREG.html]

Details for gene set RICHARDS\_NOWAKOWSKI\_SCIENCE\_2017\_NIN3\_UPREG[GSEA]

|  || Dataset | state23071 |
| Phenotype | NoPhenotypeAvailable |
| Upregulated in class | na\_neg |
| GeneSet | RICHARDS\_NOWAKOWSKI\_SCIENCE\_2017\_NIN3\_UPREG |
| Enrichment Score (ES) | -0.5858972 |
| Normalized Enrichment Score (NES) | -3.0799696 |
| Nominal p-value | 0.0 |
| FDR q-value | 0.0 |
| FWER p-Value | 0.0 |
Table: GSEA Results Summary

  

Fig 1: Enrichment plot: RICHARDS\_NOWAKOWSKI\_SCIENCE\_2017\_NIN3\_UPREG      
 Profile of the Running ES Score & Positions of GeneSet Members on the Rank Ordered List

  

| PROBE | GENE SYMBOL | GENE\_TITLE | RANK IN GENE LIST | RANK METRIC SCORE | RUNNING ES | CORE ENRICHMENT || 1 | POLE3 |  |  | 297 | 0.758 | -0.0611 | No |
| 2 | IGF2BP1 |  |  | 327 | 0.731 | -0.0559 | No |
| 3 | KLF6 |  |  | 357 | 0.708 | -0.0512 | No |
| 4 | CACYBP |  |  | 613 | 0.573 | -0.1049 | No |
| 5 | ACTG1 |  |  | 755 | 0.514 | -0.1313 | No |
| 6 | RND3 |  |  | 860 | 0.482 | -0.1490 | No |
| 7 | RHOB |  |  | 881 | 0.476 | -0.1460 | No |
| 8 | CCT4 |  |  | 999 | 0.445 | -0.1675 | No |
| 9 | HSP90AA1 |  |  | 1008 | 0.443 | -0.1620 | No |
| 10 | SACS |  |  | 1195 | 0.397 | -0.2016 | No |
| 11 | STIP1 |  |  | 1227 | 0.392 | -0.2027 | No |
| 12 | H1F0 |  |  | 1229 | 0.391 | -0.1963 | No |
| 13 | HSPA1A |  |  | 1409 | 0.356 | -0.2348 | No |
| 14 | ACTB |  |  | 1565 | 0.330 | -0.2678 | No |
| 15 | DIMT1 |  |  | 1591 | 0.326 | -0.2685 | No |
| 16 | HSPH1 |  |  | 1595 | 0.324 | -0.2638 | No |
| 17 | EFNA5 |  |  | 1624 | 0.320 | -0.2653 | No |
| 18 | ST7 |  |  | 1715 | 0.308 | -0.2825 | No |
| 19 | CHORDC1 |  |  | 1882 | 0.288 | -0.3190 | No |
| 20 | EIF1 |  |  | 2139 | 0.258 | -0.3783 | No |
| 21 | KALRN |  |  | 2182 | 0.254 | -0.3845 | No |
| 22 | KIZ |  |  | 2301 | -0.259 | -0.4095 | No |
| 23 | TP53INP1 |  |  | 2337 | -0.263 | -0.4137 | No |
| 24 | RUNX1T1 |  |  | 2458 | -0.278 | -0.4389 | No |
| 25 | MTRNR2L1 |  |  | 2471 | -0.280 | -0.4371 | No |
| 26 | IGF1R |  |  | 2700 | -0.316 | -0.4885 | No |
| 27 | NDUFAB1 |  |  | 2716 | -0.320 | -0.4868 | No |
| 28 | BROX |  |  | 2730 | -0.322 | -0.4846 | No |
| 29 | HIP1 |  |  | 2814 | -0.338 | -0.4995 | No |
| 30 | HMBOX1 |  |  | 2833 | -0.342 | -0.4982 | No |
| 31 | MFN1 |  |  | 2859 | -0.346 | -0.4986 | No |
| 32 | FIGN |  |  | 2879 | -0.351 | -0.4974 | No |
| 33 | PPP4R2 |  |  | 2989 | -0.370 | -0.5182 | No |
| 34 | NUDCD2 |  |  | 3003 | -0.373 | -0.5152 | No |
| 35 | SLC25A36 |  |  | 3080 | -0.393 | -0.5274 | No |
| 36 | LAPTM4A |  |  | 3231 | -0.436 | -0.5574 | No |
| 37 | PDZRN3 |  |  | 3276 | -0.449 | -0.5607 | No |
| 38 | PDZRN4 |  |  | 3279 | -0.450 | -0.5536 | No |
| 39 | ST8SIA4 |  |  | 3406 | -0.500 | -0.5765 | No |
| 40 | ARX |  |  | 3443 | -0.516 | -0.5767 | Yes |
| 41 | ARL4D |  |  | 3453 | -0.521 | -0.5701 | Yes |
| 42 | ASAP1 |  |  | 3484 | -0.534 | -0.5686 | Yes |
| 43 | KLHL13 |  |  | 3486 | -0.534 | -0.5598 | Yes |
| 44 | RAB5B |  |  | 3592 | -0.600 | -0.5757 | Yes |
| 45 | FBXW7 |  |  | 3629 | -0.626 | -0.5741 | Yes |
| 46 | MUM1 |  |  | 3632 | -0.629 | -0.5639 | Yes |
| 47 | BCL11B |  |  | 3656 | -0.651 | -0.5586 | Yes |
| 48 | SERINC5 |  |  | 3676 | -0.664 | -0.5521 | Yes |
| 49 | GAD1 |  |  | 3704 | -0.690 | -0.5472 | Yes |
| 50 | BMPR2 |  |  | 3715 | -0.698 | -0.5378 | Yes |
| 51 | DLX6 |  |  | 3735 | -0.711 | -0.5305 | Yes |
| 52 | NRXN3 |  |  | 3738 | -0.714 | -0.5189 | Yes |
| 53 | BCL11A |  |  | 3756 | -0.735 | -0.5107 | Yes |
| 54 | SATB1 |  |  | 3776 | -0.767 | -0.5025 | Yes |
| 55 | SOX4 |  |  | 3797 | -0.786 | -0.4941 | Yes |
| 56 | MAPK10 |  |  | 3798 | -0.787 | -0.4808 | Yes |
| 57 | CDKN1C |  |  | 3821 | -0.809 | -0.4726 | Yes |
| 58 | PFN2 |  |  | 3841 | -0.832 | -0.4632 | Yes |
| 59 | CCDC88A |  |  | 3854 | -0.848 | -0.4519 | Yes |
| 60 | SLAIN1 |  |  | 3862 | -0.855 | -0.4391 | Yes |
| 61 | CADPS |  |  | 3877 | -0.894 | -0.4275 | Yes |
| 62 | RBP1 |  |  | 3878 | -0.898 | -0.4123 | Yes |
| 63 | CHD7 |  |  | 3880 | -0.904 | -0.3972 | Yes |
| 64 | MAF |  |  | 3893 | -0.930 | -0.3844 | Yes |
| 65 | SP9 |  |  | 3898 | -0.942 | -0.3695 | Yes |
| 66 | FOS |  |  | 3911 | -0.966 | -0.3561 | Yes |
| 67 | KCNQ1OT1 |  |  | 3916 | -0.988 | -0.3404 | Yes |
| 68 | DCLK2 |  |  | 3945 | -1.043 | -0.3297 | Yes |
| 69 | SOX6 |  |  | 3956 | -1.081 | -0.3139 | Yes |
| 70 | NEDD4L |  |  | 3960 | -1.090 | -0.2962 | Yes |
| 71 | SOX11 |  |  | 3977 | -1.147 | -0.2807 | Yes |
| 72 | TCF4 |  |  | 3984 | -1.173 | -0.2624 | Yes |
| 73 | DPYSL3 |  |  | 4012 | -1.349 | -0.2462 | Yes |
| 74 | PNRC1 |  |  | 4022 | -1.397 | -0.2248 | Yes |
| 75 | LSAMP |  |  | 4028 | -1.442 | -0.2016 | Yes |
| 76 | DLX1 |  |  | 4041 | -1.558 | -0.1782 | Yes |
| 77 | DLX2 |  |  | 4055 | -1.704 | -0.1526 | Yes |
| 78 | PLXNA4 |  |  | 4060 | -1.752 | -0.1240 | Yes |
| 79 | SNTG1 |  |  | 4086 | -2.546 | -0.0871 | Yes |
| 80 | DLX5 |  |  | 4090 | -2.619 | -0.0435 | Yes |
| 81 | DCX |  |  | 4091 | -2.671 | 0.0017 | Yes |
Table: GSEA details [plain text format]

  

Fig 2: RICHARDS\_NOWAKOWSKI\_SCIENCE\_2017\_NIN3\_UPREG: Random ES distribution      
 Gene set null distribution of ES for **RICHARDS\_NOWAKOWSKI\_SCIENCE\_2017\_NIN3\_UPREG**
